# An acceptability and safety study of the Duet^® ^cervical barrier and gel delivery system in Zimbabwe

**DOI:** 10.1186/1758-2652-13-30

**Published:** 2010-08-05

**Authors:** Elizabeth T Montgomery, Cynthia Woodsong, Petina Musara, Helen Cheng, Tsungai Chipato, Thomas R Moench, Freya Spielberg, Ariane van der Straten

**Affiliations:** 1 Women's Global Health Imperative, RTI International, San Francisco Project Office, San Francisco, CA, USA; 2 International Partnership for Microbicides, Paarl, South Africa; 3 University of Zimbabwe-University of California San Francisco Research Collaborative Programme in Women's Health, Harare, Zimbabwe; 4 ReProtect, Inc., Baltimore, MD, USA; 5 Center for AIDS Prevention Studies, Department of Medicine, University of California San Francisco, San Francisco, CA, USA

## Abstract

**Background:**

Adherence problems with coitally dependent, female-initiated HIV prevention methods have contributed to several trials' failure to establish efficacy. Continuous use of a cervical barrier with once-daily cleaning and immediate reinsertion may simplify use for women and improve adherence. We assessed the acceptability and safety of precoital and continuous use of the Duet^®^, a cervical barrier and gel delivery system, in Zimbabwean women.

**Methods:**

Using a two-arm crossover design with a parallel observation group, we randomized 103 women in a **2:2:**1 ratio: (1) to use the Duet continuously for 14 days, followed by a minimum of seven days of washout and then 14 days of precoital use; (2) to use the same Duet regimens in reverse order; or (3) for observation only. Women were aged 18 to 40 years; half were recruited from a pool of previous diaphragm study participants and the other half from the general community. Acceptability and adherence were assessed through an interviewer-administered questionnaire at each of two follow-up visits. Safety was monitored through pelvic speculum exams and report of adverse events.

**Results:**

The proportion of women who reported consistent Duet use during sex was virtually identical during continuous and precoital regimens (88.6% vs. 88.9%). Partner refusal was the most common reason cited for non-use during sex in both use regimens. Not having the device handy was the most common reason cited for non-daily use (in the continuous regimen). Most women were "very comfortable" using it continuously (86.3%) and inserting it precoitally (92.8%). The most favoured Duet attribute was that it did not interfere with "natural" sex (55%). The least favoured Duet attribute was the concern that it *might *come out during sex (71.3%). No serious adverse events were reported during the study; 57 participants reported 90 adverse events classified as mild or moderate. There were no statistically significant differences in: (1) the proportion of women reporting adverse events; (2) the severity of events among those using the Duet and observational controls; or (3) event severity reported during each regimen use period.

**Conclusions:**

In this study, the Duet was found to be acceptable and safe when inserted precoitally or used continuously for 14 days. Assignment to use of the Duet continuously did not increase adherence to the Duet during sex. Future HIV prevention trials should evaluate use of the Duet (precoitally and continuously) with promising microbicide candidates.

## Background

Female-initiated methods to prevent HIV, including microbicides, intravaginal rings and cervical barriers, are currently being investigated in clinical trials to address the disproportionate burden of HIV in women, particularly in the highest prevalence regions, such as sub-Saharan Africa [1]. Such technologies would provide additional disease prevention options to women and couples, and might be particularly beneficial in partnerships where male condom use is not feasible.

Thus far, 11 trials of microbicide candidates and one trial of the diaphragm with a lubricant gel were unable to detect a significant reduction in HIV acquisition in women [2]. One important reason that trials of these investigational female-initiated products may have been unable to detect a significant protective effect is the lower-than-anticipated adherence levels reported by trial participants, which may have diluted the power to measure the effectiveness of the intervention had there been true biological efficacy [2-4]. Unlike male circumcision, these interventions are user dependent, and optimal adherence necessitates the insertion of a device and/or applicator of gel shortly before sex, which may be difficult for practical, cultural or other reasons.

One potential strategy to simplify use and increase adherence to female-initiated methods is to make them coitally independent. Continuous use of cervical barriers has been promoted as such a strategy: products are always worn and only removed once daily for cleaning, and then immediately reinserted. There are limited data on the safety and acceptability of this approach. Two pilot studies in Madagascar promoted continuous use of the diaphragm for four or eight weeks among sex workers. In one study, 92% of respondents reported being somewhat comfortable or comfortable with this technique, although at 20% of visits, the participants reported multiple daily insertions of the diaphragm, indicating that the continuous-use protocol was not followed [5]. In the other study, 88% of women reported continuous diaphragm use in the previous day at Week 1, and this usage increased to 93% by Week 8 [6]. Although primarily focused on the acceptability and feasibility of continuous use of the diaphragm among the study populations, these studies also monitored safety outcomes and reported that continuous use appeared safe [5,6].

In this study, we aimed primarily to assess the acceptability of the Duet^® ^cervical barrier (with single-use 10 g BufferGel sachets) when inserted precoitally or used continuously in an African population, and to simultaneously monitor safety using a parallel observation group. The Duet is a diaphragm-like device designed by ReProtect, Inc. (Baltimore, MD, USA) to act as both a cervical barrier and a gel delivery system capable of delivering gel on both its cervical and vaginal lumen sides. Its single-size design is intended to fit most women and does not require medical fitting. It was originally designed for use with BufferGel^®^, an acid-buffering gel that has been shown to have microbicidal activity *in vitro *and in animal models [7-9], and to be safe for human use and effective in preventing pregnancy when used with a diaphragm, although ineffective on its own in preventing HIV [10-13]. The functional performance, safety and acceptability of the Duet (prefilled with BufferGel) was first investigated in the United States and the Dominican Republic among 30 couples who used the device during sex twice in one week [14]. In that study, the Duet was found to be safe and acceptable, and it was concluded that further research into the product as a potential disease prevention method was warranted [14].

Importantly, the Duet can be used with other vaginal gels, including alternative microbicide candidates, and a combination product could theoretically provide enhanced protection against diseases through the additive effect of a physical barrier and gel. The results from this study are important to consider within this context.

## Methods

### Study setting and population

The study was conducted from December 2008 to May 2009 at a research site in Epworth, a peri-urban township within the municipal district of Harare, the capital city of Zimbabwe. Inclusion criteria included: being 18 to 40 years of age; being sexually active (one or more acts of vaginal sex per week for the three months prior to enrolment); being non-pregnant and on a highly effective form of contraception; having no genital abnormalities or symptoms; being able to successfully insert and remove the Duet within three attempts; and being willing and able to provide informed consent. Women with abnormal menstrual cycles were excluded.

HIV-positive status was not an exclusion criterion, but women had to be physically healthy in the judgment of the study clinicians, with no history of any serious acute, chronic or progressive disease, and no known allergy to latex. As a secondary objective, the study aimed to evaluate how previous diaphragm experience might impact the acceptability of the Duet worn continuously and precoitally. Thus, half of the enrolled study sample participants were purposively recruited from a randomly selected list of former intervention-arm participants in the Methods for Improving Reproductive Health in Africa (MIRA) trial (a Phase III trial of the effectiveness of the diaphragm in preventing HIV), who lived in the Epworth area and were within the eligible age range [15]. The remaining participants for this study were not former MIRA participants and were either recruited by the study outreach worker at community venues or walked in to the research centre.

### Study design

**Figure 1:**
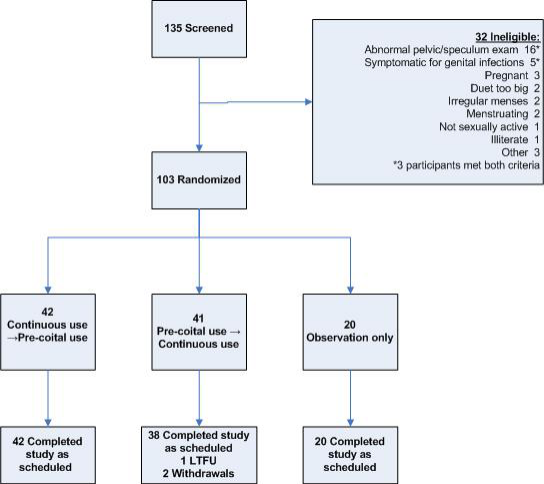
**Study profile**.

The study was an open-label, two-arm crossover study with a parallel observation group randomized in a **2:2:**1 ratio (see Figure 1). Forty percent of women (the continuous→precoital group) were randomized to wear the Duet continuously for 14 days, followed by a minimum of seven days of washout, and 14 days of precoital use; 40% of women were randomized to the reverse-order regimen (the precoital→continuous group). To better inform the safety evaluation, the remaining 20% were assigned to an observation-only arm. Duet-use regimens and study visits were scheduled to avoid menses. All groups received male condoms, and safe sex and risk reduction counselling.

### Study products

**Figure 2:**
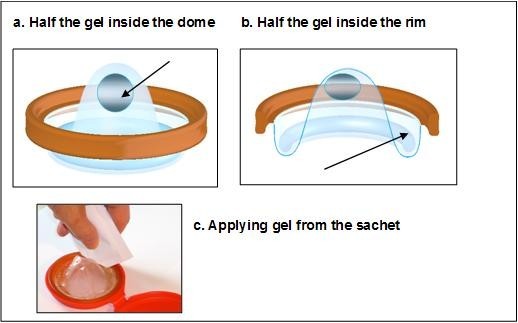
**Diagram of Duet and method for gel application**.

The Duet is similar in structure to a diaphragm, but with a hard rim and dome. However, the Duet has a wider rim and a reservoir surrounding the rim at the base of the dome that acts as a gel collection area (see Figure 2). BufferGel was provided in single-use 10 g sachets (similar to condiment packaging). Gel was applied on both the vaginal and cervical sides of the Duet prior to insertion. Prior research has been conducted on the acceptability of the Duet as a menstrual collection device [16], and contraceptive efficacy studies are planned. However, participants were advised that this study was investigating the product's potential use for delivery of gels for HIV and sexually transmitted infection prevention.

### Study procedures

At screening, participants gave oral consent to complete a short, interviewer-administered eligibility questionnaire, and participants who met initial inclusion and exclusion criteria were then asked to provide written informed consent for study procedures. A urine specimen was collected for pregnancy testing, and participants were interviewed about their demographic and clinical history. Non-pregnant participants were given a pelvic speculum exam to rule out genital abnormalities, signs or symptoms, and two swabs were collected from the vaginal fornices for assessment of bacterial vaginosis (BV). One was used for a rapid point-of-care assay (BVBlue^®^, Gryphus Diagnostics, Knoxville, TN, USA), and the second for assessment of BV using both Amsell's criteria and Nugent scoring [17,18].

BV and all other genital signs and symptoms were treated syndromically, according to Zimbabwe National Syndromic Management guidelines [19]. Clinicians demonstrated how to load gel in and on the Duet from the sachet, and inserted it in a translucent pelvic model. Participants then practiced the insertion and removal of the Duet on themselves. All potential participants were able to successfully insert and remove the Duet within three attempts.

The randomization scheme was based on a permuted block design with randomized blocks of sizes 5 and 10. Participants randomized to the continuous→precoital group initiated the continuous-use regimen immediately and inserted the Duet while in the clinic. They were given 14 sachets of gel, the Duet plastic case, condoms, and an illustrated, local language (Shona) or English Duet instruction sheet for continuous use.

Participants in the precoital→continuous group were given a Duet to insert within one hour prior to sex, as well as a supply of gel sachets commensurate with their reported coital frequency, male condoms, a Duet plastic case, and illustrated instruction sheet for precoital use. Precoital use specified that the Duet should be inserted within one hour prior to sex, and removed no sooner than one hour and no later than 24 hours postcoitus. All participants were given male condoms.

Participants in the Duet-use groups were also provided with panty liners in case of gel leakage, and a bar of mild soap to clean the Duet. Following randomization, participants completed an interviewer-administered baseline acceptability and adherence questionnaire, received safe sex and risk reduction counselling, and were scheduled for their first follow-up visit 14 days after enrolment (a window of about seven days was allowable). Participants were encouraged to come to the clinic at any time if they required clinical attention, counselling or resupply of gel or condoms.

At the first follow up, participants completed an interviewer-administered adherence questionnaire. Next, a clinician conducted a clinical interview and pelvic speculum exam to identify any adverse events (AEs), and to obtain vaginal swabs for BV diagnosis. Women with genital signs or symptoms of infection were treated syndromically, as described, and scheduled to return to the clinic within seven to 14 days for evaluation. Participants in the Duet-use groups received crossover counselling to review the procedures and dates for switching their use regimen following menses and washout. Used and unused gel sachets were returned and inventoried, and new gel sachets and instructions were issued in accordance with the subsequent-use regimen.

All participants received condoms and risk reduction counselling, and completed an interviewer-administered acceptability questionnaire. After reviewing locator information, participants were reimbursed for their transport and time, and scheduled to return to the clinic approximately one month later. A study outreach worker contacted Duet-use participants at home within two days of the target date for crossing over to the new Duet-use regimen to ensure that change in regimen occurred correctly.

The second and final follow-up visit followed the same sequence of procedures as the first follow up, except for crossover counselling. All study products (Duet, used and unused gel sachets) were permanently collected and inventoried, and participants were discontinued from the clinical portion of the study. Updated locator information and willingness to be contacted within 60 days to participate in a qualitative component of the study (data to be presented in a separate paper) were solicited from intervention group women.

### Measures and statistical analysis

We assessed the acceptability, adherence and safety of the Duet for use among African women when used continuously for 14 days or inserted precoitally for 14 days.

#### Acceptability measures

Among intervention group women, the comfort of wearing the Duet during each regimen was assessed at the visit immediately following that regimen. At the acceptability assessment at the final visit, the following information was obtained: measures that assessed willingness to use the Duet if one perceived oneself to be at risk for HIV; comfort using the gel sachets and loading gel in the device; preferences for the continuous versus precoital regimen for the participant and her perception of her partner's preference; and the characteristics most women found favourable and unfavourable about Duet use for themselves and their partners (i.e., the appearance of the Duet and the effect on sex). After we determined that no differences were observed in acceptability measures by the Duet group assignment (i.e., order-effect), we combined acceptability results for both Duet-use groups.

#### Adherence measures

We computed the mean, median and range for the number of days the Duet was reported to be used during the continuous-use regimen, and the number of sex acts during both regimens. We evaluated adherence to the assigned regimen by computing the proportion of days (100%, ≥ 80%) that the Duet was worn during the continuous-use regimen and the proportion of sex acts (100%, ≥ 80%) during which the Duet was worn during both the continuous-use and precoital-use regimens. Condom use adherence and the number of times the Duet came out on its own were tabulated. We also assessed the reasons for sub-optimal adherence among those who were imperfect users. Finally, to explore the potential influence of previous cervical barrier use on adherence and acceptability to the Duet, we used chi-square statistics to compare the proportion of women reporting key measures by prior diaphragm experience.

#### Safety measures

These were based on a tabulation of participants' self-reports of AEs and clinician classification of those events, abnormal physical findings, the results of the point-of-care BV test (Amsell's criteria and Nugent score not shown), and reports of emotional problems or social harms. For AEs, we computed the number of individuals experiencing events, the number of events observed, and the number of events per 100 person weeks of observation, by group. This study was not powered to identify statistical differences between groups. However, for illustrative purposes, we compared: (1) the proportion of women experiencing AEs among those using the Duet versus observational controls using Fisher's exact test statistics; and (2) the proportion of women reporting AEs during the continuous-use versus precoital-use regimens. Because participants participated in each regimen, the data were treated as paired data and analyzed using a McNemar's test.

The study was carried out under the principles outlined in the Declaration of Helsinki and was reviewed and approved by Institutional Review Boards at RTI International (USA) and the Medical Research Council of Zimbabwe. The Medicines Control Authority of Zimbabwe provided regulatory approval for the conduct of the study.

## Results

### Study sample

Table 1 presents the baseline characteristics of the 103 women enrolled in the study (42 continuous→precoital group, 41 precoital→continuous group, 20 observation-only group), who had a mean and median age of 30 years, were all married, and mostly Shona (66%), the majority tribal group in Zimbabwe. All women had at least one child, and the majority (63%) had more than two children. Use of a highly effective contraceptive was an inclusion criterion; most women (66.7%) were using oral contraceptives, while 28.4% were using injectables.

**Table 1:** Baseline characteristics of study sample, overall and by group assignment

Characteristic	Total (n = 103)		Continuous→precoital (n = 42)		Precoital→continuous (n = 41)		Observation (n = 20)	
	**mean**	**median**	**mean**	**median**	**mean**	**median**	**mean**	**median**
**Age**	30.2	30.0	29.7	29.0	30.8	30.0	29.9	31.0
**No. partners, past 3 mos**.	1.0	1.0	1.0	1.0	1.0	1.0	1.0	1.0
	**n**	**%**	**n**	**%**	**n**	**%**	**n**	**%**
	
**Ethnicity**								
Shona	67	65.7	28	66.7	27	65.9	12	63.2
Other	35	34.3	14	33.3	14	34.2	7	36.8
**Relationship status**								
Married	102	100.0	42	100.0	41	100.0	19	100.0
**Parity**								
1-2	39	38.2	22	52.4	13	31.7	4	21.0
3 or more	63	61.7	20	47.6	28	68.3	15	78.9
**Current contraception**								
Long-term contraceptives	5	4.9	1	2.4	3	7.3	1	5.3
Injectables	29	28.4	12	28.6	14	34.2	3	15.8
Oral contraceptives	68	66.7	29	69.1	24	58.5	15	79.0
**Ever treated for RTI/STI, lifetime**								
Yes	15	14.7	8	19.1	5	12.2	2	10.5
**Any symptoms, past 3 mos**.								
Yes	14	13.7	6	14.3	6	14.6	2	10.5
**BV status at baseline**								
Positive	19	19.2	12	28.6	4	10.8	3	15.0
**Ever used male condoms**								
Yes	83	80.6	32	76.2	32	78.1	19	95.0
**Male condom use, past 3 mos**.								
All of the time	8	9.5	1	3.0	4	12.5	3	15.8
Sometimes	35	41.7	16	48.5	9	28.1	10	52.6
Never	41	48.8	16	48.5	19	59.4	6	31.6
**Ever used diaphragm, past 3 mos**.								
Yes	12	11.7	4	9.5	5	12.2	3	15.0

For sexual risk behavior, the mean and median number of sexual partners in the past three months was one, and less than 15% had ever been treated for a sexually transmitted infection (STI), although 14% also reported having some vaginal symptoms in the past three months and almost one-fifth (19.2%) were BV positive per rapid assay (and asymptomatic, thus not treated). While 81% reported having *ever *used male condoms in their lifetime, only half (51.2%) reported using male condoms in the past three months.

Twelve of the 52 former MIRA participants (23.1%) had used a diaphragm in the past three months, but none of these women cited the diaphragm as a current contraceptive method. Randomization groups were largely similar for all measured baseline characteristics; although the parity distribution for women in the continuous→precoital group was lower than those in the other two groups, these women were also more likely to be BV positive (per rapid assay) at baseline. Women in the observation group were slightly more likely to report ever having used male condoms. All enrolled participants were able to wear the Duet device; only two of the 135 women screened were unable to wear the Duet due to an unsuitable fit (see Figure 1).

### Adherence to Duet-use regimen

We examined adherence among Duet users by regimen. When participants were assigned to use the Duet continuously, they wore it all day or at least part of the day for, on average, 13.3 of the target 14 days (median 14, range 7-14; see Table 2). Overall, the majority (81%) of women reported that they wore the Duet for all of their continuous-use days, and 90% used it for at least 80% of the days they were instructed to use it (see Table 2). During the continuous-use regimen, each woman reported an average of 10 sex acts overall (mean 10.2, median 10) for the 14-day period, and 88.6% reported using the Duet every time they had sex, while 63.3% reported using condoms every time.

**Table 2:** Adherence to Duet use among intervention group participants (n = 83), by regimen

	Continuous regimen (n = 80)		Precoital regimen (n = 82)	
**Duet adherence**	**mean**	**med (range)**	**mean**	**med (range)**
	
Days Duet worn	13.3	14.0 (7-14)	NA	NA
Number of sex acts	10.2	9.0 (2-42)	10.5	10.0 (2-42)
	
	**n**	**%**	**n**	**%**
	
Duet regimen adherence				
Worn at least 80% of days	71	89.9	NA	NA
Worn 100% of days	64	81	NA	NA
Duet adherence during sex				
Worn at least 80% of sex acts	74	93.7	77	93.9
Worn 100% of sex acts	70	88.6	73	89
Ever used Duet precoitally during continuous regimen	1	1.3	1	1.3
Duet ever came out on its own	2	2.5	0	0
Mean/median number of times Duet came out on its own*	1.5	1.5	0	0
**Reasons for non-adherence**				
Main reason Duet not used during sex				
*Used the Duet every time for sex*	69	87.3	74	90.2
*Partner refused to use it*	4	5.1	3	3.7
*Did not have Duet with her/forgot to use it*	1 report each	2.6	2 reports each	4.8
*Discomfort/having menses/did not have time to insert*	1 report each	3.9	0	0
*Other*	2	2.5	1	1.2
Main reason Duet not used every day				
*Used the Duet every day*	62	78.5	NA	NA
*Did not have the Duet with her *	5	6.3	NA	NA
*Partner refused to use it*	3	3.8	NA	NA
*Discomfort/did not have sex/having menses/other*	2 reports each	10.0	NA	NA
*Forgot to use it*	1	1.3	NA	NA
**Condom adherence**				
Worn at least 80% of sex acts	52	65.8	53	65.4
Worn 100% of sex acts	50	63.3	53	65.4

*Denominators and proportions exclude missing data for n = 4 for continuous-use adherence and n = 1 for precoital-use adherence.

During their precoital regimen, women reported a similar number of sex acts for the 14-day period (mean 10.5, median 10), and the overall proportion of women who reported consistent Duet use during sex was virtually identical to that during the continuous-use regimen (88.9%). Reported condom use during sex was similar to the continuous-use regimen, with roughly two-thirds of women reporting condom use for 100% of sex acts. Reports of crossover contamination and involuntary Duet removal were rare (see Table 2). Compared to those in the Duet groups, more women in the observation group (80%) reported consistent male condom use, and they had only slightly fewer sex acts (mean 9.3, median 8.5) during the 14-day periods assessed.

During both regimen periods, we asked women their main reasons for *not *using the Duet every day or during every act of sex, and supplied some categorical responses, as well as an open-ended response. Although the majority (range 79%-90%; see Table 2) indicated that they *had *used it every time, partner refusal was the most commonly cited reason for non-adherence during sex in both regimen periods. Reasons for not using the Duet every day during the continuous regimen were more varied: the most common reason participants cited was that they did not have it with them. Partner refusal, discomfort, having menses, and non-use because they did not have sex (demonstrating protocol misunderstanding) were also mentioned (see Table 2).

### Acceptability

Overall, 86.3% of women reported feeling "very comfortable" wearing the Duet continuously for 14 days, while 92.8% reported the same for the precoital regimen (see Table 3). Just over half (51.4%) of the participants reported a preference for the precoital regimen, 10% felt they were equivalent, and 39% preferred continuous use. Women's perceptions of their partner's preferences for continuous or precoital use mirrored their own preferences. A majority (85%) of women reported that they would "definitely" use the Duet if they thought they were at risk for HIV and it could help protect them (see Table 3).

**Table 3:** Acceptability of the Duet among intervention group participants (n = 83)

	**#**	**%**
	
**Very comfortable wearing the Duet continuously**	69	86.3
Somewhat comfortable	10	12.5
Not at all	1	92.8
**Very comfortable inserting and removing the Duet before and after each act of sex**	77	92.8
Somewhat comfortable	5	6.0
Not at all	1	1.2
**Definitely would use Duet if I thought I was at risk for HIV and Duet could protect me**	68	85.0
I maybe would use the Duet	11	13.8
I maybe would not use the Duet	1	1.3
I definitely would not use the Duet	0	-
**Very comfortable opening the gel sachet**	79	98.8
Somewhat comfortable	1	1.3
Not at all	0	0
**Very comfortable loading the gel into the Duet**	97.5	80
Somewhat comfortable	2.5	2
Not at all		
**Preferred regimen**		
Continuous	31	38.8
Precoital	41	51.3
Same	8	10.0
**Preferred regimen of partner**		
Continuous	30	37.5
Precoital	41	51.3
Same	9	11.3
**Most favourable characteristic of Duet for participant (top 3)**		
Duet does not interfere with normal/natural sex	44	55.0
The Duet is reusable	15	18.8
You can put the Duet in yourself	9	11.3
**Least favourable characteristic of Duet for participant (top 3)**		
The Duet might come out during sex	57	71.3
Duet might change the feeling of sex for you	11	13.8
Duet might change the feeling of sex for him	10	12.5
**Characteristic participant perceived as most favourable for partner (top 3)**		
Duet does not interfere with normal/natural sex	56	68.8
The Duet is reusable	10	12.5
You can put the Duet in yourself	10	12.5
**Characteristic participant perceived as least favourable for partner (top 3)**		
The Duet might come out during sex	36	70.0
Duet might change the feeling of sex for him	13	16.3
He might feel Duet in your body during sex	5	6.3

Women were provided with a list of Duet attributes, and asked to indicate which they found the most and least favourable (see Table 3). The top three attributes of the Duet that most participants cited were that it doesn't interfere with "normal/natural sex" (55%), it is reusable (18.8%), and it is inserted by the woman herself (11.3%). Women ascribed their partner's preferences to the same three characteristics, although greater emphasis was placed on the first attribute: that it does not interfere with "natural" sex.

The three *least favourable *characteristics of the Duet for women were all hypothetical attributes: that it might come out during sex (71.3%); and that it might change the feeling of sex either for women themselves (13.8%) or their partners (12.5%). Here again, women's perceptions of their partners' attitudes towards the least favoured characteristics were similar to their own. Women also reported that their partners would disfavour the Duet if they could feel it during sex, and this ranked as potentially more disfavourable than a decrease in the women's own sexual pleasure.

### Previous diaphragm experience

We compared responses to key adherence and acceptability measures between those who had previously used a diaphragm and those who had not ("diaphragm naïve") using Fisher's exact tests. No differences were statistically significant; however, there was a trend towards a preference for precoital use and better adherence to the precoital-use regimen among diaphragm-experienced women (see Table 4).

**Table 4:** Adherence and acceptability of the Duet, by previous diaphragm experience (n = 83)

	Dia-naïve ppts (n = 41)		Dia-experienced ppts (n = 42)		p value*
**Continuous regimen adherence**	**n**	**%**	**n**	**%**	
Duet regimen adherence					
Worn at least 80% of days	37	94.9	34	85.0	*0.26*
Worn 100% of days	33	84.6	31	77.5	*0.57*
Duet adherence during sex					
Worn at least 80% of sex acts	38	97.4	36	90.0	*0.36*
Worn 100% of sex acts	36	92.3	34	85.0	*0.48*
**Precoital regimen adherence**					
Duet adherence during sex					
Worn at least 80% of sex acts	36	87.8	41	100.0	*0.06*
Worn 100% of sex acts	35	85.4	38	92.7	*0.48*
**Acceptability**					
Very comfortable wearing the Duet continuously	34	85.0	35	87.5	*0.74*
Somewhat comfortable	6	15.0	4	10.0	
Not at all	0	-	1	2.5	
Very comfortable inserting and removing the Duet before and after each act of sex	37	90.2	40	95.2	*0.51*
Somewhat comfortable	3	7.3	2	4.8	
Not at all	1	2.4	0	-	
Willingness to use Duet if you thought you were at risk for HIV and Duet could protect you					*1.00*
I definitely would use the Duet	34	85.0	34	85.0	
I maybe would use the Duet	5	12.5	6	15.0	
I maybe would not use the Duet	1	2.5	0	-	
I definitely would not use the Duet	0	-	0	-	
**Preferred regimen**					*0.13*
Continuous	20	50.0	11	27.5	
Precoital	17	42.5	24	60.0	
Same	3	7.5	5	12.5	
**Preferred regimen of partner**					*0.34*
Continuous	18	45.0	12	30.0	
Precoital	19	47.5	22	55.0	
Same	3	7.5	6	15.0	

*P value for the comparison between diaphragm-naïve and diaphragm-experienced participants, based on Fisher's exact test

### Safety

#### Adverse events

During the course of the study, no serious AEs occurred; 57 participants reported 90 AEs that were classified as mild or moderate (see Table 5). There were no statistically significant differences in: (1) the proportion of women reporting AEs (overall and reproductive tract or urinary tract related); (2) the severity of events reported among those using the Duet and those in the observation group; or (3) the severity of events reported during each regimen use period. Similarly, the distribution of relatedness to study product was not significant (p >0.05) between the two Duet regimens (not applicable for observation group).

**Table 5:** Total number of women experiencing adverse events (n), number of AEs (#), and number of AEs per 100 person weeks, by regimen

	Continuous regimen (n = 80)*				Precoital regimen (n = 83)*				Observation (n = 20)*		
	
	n	#	#/100 woman weeks	*p^1^*	N	#	#/100 woman weeks	*p^2^*	n	#	#/100 woman weeks
**Total**	28	38	23.8	*0.60*	32	39	23.5	*1.00*	11	13	16.3
Reproductive tract (RT) or urinary tract (UT) related	12	15	9.4	*0.42*	8	10	6.0	*1.00*	4	4	5.0
											
**Severity**											
**Mild**	20	23	14.4	*0.86*	22	27	16.3	*0.62*	10	11	13.8
RT/UT related	5	6	3.8	*0.75*	7	8	4.8	*1.00*	3	3	3.8
**Moderate**	11	15	9.4	*1.00*	10	12	7.2	*0.23*	2	2	2.5
RT/UT related	7	9	5.6	*0.07*	1	2	1.2	*1.00*	1	1	1.3
**Severe**	0	0	-	*-*	0	0	-	*-*	0	0	-
RT/UT related	0	0	-	*-*	0	0	-	*-*	0	0	-
**Relatedness to study products**											
Definitely	0	0	-	*-*	0	0	-	*-*	-	0	-
Probably	4	4	2.5	*0.73*	6	6	3.6	*-*	-	0	-
Possibly	6	9	5.6	*0.75*	4	4	2.4	*-*	-	0	-
Probably not	4	4	2.5	*1.00*	4	4	2.4	*-*	-	0	-
Not related	14	21	13.1	*0.21*	21	25	15.1	*-*	-	13	-

* Duet use participants were followed for up to 14 days per regimen. Observation group participants were followed for up to 28 days.

^1 ^P value for the comparison of the proportion of intervention group women reporting an event during the continuous vs. precoital regimen periods (McNemar's test)

^2 ^P value for the comparison of the proportion of women reporting an event between those in an intervention group vs. observational controls (Fisher's exact test)

In total, 31 events (among 28 women) were classified as related to study products: 13 "possibly", 10 "probably", eight "probably not", and zero "definitely" (see Table 5). The majority of related events were reproductive tract or urinary tract related. However, a few participants reported physiological/neurological discomfort associated with the squatting position that some women used for inserting and removing the Duet (i.e., numbness or pain in hips).

Three individuals had abnormal physical exam findings at each of two follow-up visits (data not shown). One of these women had persistent cervical erythema following her precoital-use regimen that necessitated study discontinuation. The remaining five cases were noted after the continuous-use regimen and included findings of abnormal discharge (two women), candidiasis (one), cervical ectopy (one) and suspected pelvic inflammatory disease (one). No epithelial disruptions were identified.

The baseline distribution of BV-positive BV Blue results was unevenly distributed by arm, with the continuous→precoital group having almost twice the proportion as the other two groups (see Table 1). In the follow-up period, across all groups, 19 BV Blue-positive results were observed among 15 individuals (data not shown). Seven of these 19 BV-positive results during follow up were among women who were negative for BV at enrolment, and this was distributed evenly as two cases per group (one individual in the continuous→precoital group was positive at both follow-up visits).

#### Emotional problems and social harms

A minority of individuals also reported emotional problems caused by the Duet: during the continuous regimen, four women (5%) reported "fear", one reported verbal abuse from the partner, and one reported partner complaints about the gel. During the precoital regimen, four women reported "fear", two cited verbal abuse from partners, and one reported "shame".

## Discussion

This is the first study to assess acceptability and safety of the Duet used with a vaginal gel for 14 days continuously or precoitally in an African setting. Although BufferGel alone and the diaphragm used with an inactive lubricant have not been shown to be effective in preventing HIV in this setting [12,15], the combined effectiveness of a cervical barrier, such as the Duet and an effective microbicide, has not yet been tested. Thus, the safety and acceptability results reported here may inform future trials of products such as these, used independently or in combination.

Overall, women were very comfortable wearing the Duet continuously or inserting it precoitally, and the majority indicated that they would use it in the future if it were shown to be effective in preventing HIV and they perceived themselves to be at risk. Since the Duet and gel were used together, it is difficult to disentangle attitudes towards the device alone versus the gel alone. For example, when responding to questions about "Duet use", women may have automatically incorporated "gel use" in their assessment, or may have been referencing only the device. The majority of women reported being "very comfortable" with the functional aspects (opening and loading) of the single-use gel sachets, and thus, it is likely reasonable to assume that favourable attitudes towards the Duet applied to impressions of both the cervical barrier and the gel application process, if not also the gel itself.

Women who were former MIRA participants and had previous diaphragm experience (used precoitally) appear to have a preference for precoital-use regimen for the Duet, although the difference was not statistically significant at the p < 0.05 level. Further analysis is planned to more specifically examine the characteristics of women who favoured continuous versus precoital use. Mirroring the preferences for one regimen over another, women who were diaphragm experienced were more consistent users of the Duet when asked to use it precoitally, although again, the difference was not statistically significant. This study may have been underpowered to detect significantly reduced uptake of continuous use of this similar prevention technology because of previous familiarity with precoital insertion. However, because so few women in the region have been exposed to these technologies, this should not pose a substantial barrier to future efforts to promote or introduce continuous use of a female-initiated HIV prevention method. While it is promising that more than 80% of women overall were reportedly willing and able to use the Duet continuously during their 14-day use period, in this study the proportion of sex acts with a Duet *in situ *was almost identical for each use regimen. Thus, assignment to continuous use did not result in higher adherence during sex, when exposure to HIV or STIs may occur.

One of the previously described Madagascar pilot studies recently reported that continuous diaphragm use was associated with four times higher odds of adherent diaphragm use during sex [20]. However, these findings were based on a subgroup analysis of participants who were classified as "continuous users" using an imputed composite variable created through qualitative and quantitative data triangulation. Also, the study population had at least four monthly sexual partners, compared with our monogamous, married population [20]. In contrast, we examined Duet adherence during sex, using an intent-to-treat approach based on group assignment. Of note, among the subgroup of women (n = 64) who reported full adherence during the continuous use regimen, 97% of sex acts were protected by Duet (as compared with 89% overall). It is unknown how promotion of continuous use and its effect on adherence would change (in either direction) with longer study duration or with a larger sample size that would provide greater power to detect differences by group.

More than half the participants felt that the most favoured characteristic of the Duet is that it does not interfere with "normal/natural" sex, indicating that using the Duet during sex did not change the feeling of sex, an often cited complaint about male condoms [21,22]. Perceptions of the potential for diminishing sexual pleasure for either the participant or her partner were likewise important fears to participants, as were concerns that the device might come out during sex or that the partner may touch the device with his penis, presumably because these instances would interrupt the sex act or sexual pleasure. Sexual pleasure has been discussed as a critical consideration in microbicide and cervical barrier acceptability research because the introduction of a gel could alter the "natural" feeling of sex. Women may find that a gel increases their pleasure or reduces their pain, although they may be concerned that partners will find the lubrication to be unfavourable [23-25].

Women's top three reasons for liking or disliking the Duet were virtually identical to their perceptions of their male partners' attitudes. While we did not separately assess men's and women's perspectives, a growing body of literature, including data from the MIRA study, supports the interpretation that the male partners' acceptability of the Duet may have influenced women's own acceptability and use of the device [25-28]. Further analysis of the study's qualitative interviews with men and women will explore these issues in greater depth.

In this study, the Duet appeared safe, when used both continuously and precoitally, for 14 days. This study relied on clinical observation and self-report of adverse events and did not include colposcopic examinations. The nature and quantity of events observed is what was expected for a study of this size and duration in this setting. Although not statistically significant, more events were observed in the intervention groups, and more gynecological events were classified as "moderate" in the continuous-use regimen, but this may be attributed to women's heightened awareness and more frequent contact with their genitalia when using a product daily (compared to non-use), and when using a product continuously as compared with just during sex.

In the North American-based Duet study, the Duet was also concluded to be safe, although the authors recommended ongoing monitoring of abdominal and genital pain in future studies, and attention to peeling or disruption of the cervical epithelium [14]. In this study, no epithelial disruptions were observed on speculum exam, although one participant experienced persistent cervical erythema and was discontinued at her first follow-up visit. Several participants initially reported pain or discomfort upon inserting, removing and wearing the Duet. Although the majority of these problems resolved with counselling on proper placement or practice, these issues should continue to be monitored in future studies.

In the MIRA study, participants' reports of problems associated with diaphragm use decreased substantially after the first one or two follow-up visits with improving skills and counselling from clinic staff [29]. Unlike the contraceptive diaphragm, the Duet is a single-size device designed for use without medical fitting. Our results confirm and extend those of Ballagh *et al *in indicating that the Duet provides an appropriate fit for most women, and thus may be appropriate for over-the-counter use [14].

This study has several strengths and limitations. First, we aimed to assess the acceptability and safety of two Duet-use regimens, and both preferences and biological susceptibility to reproductive tract-or urinary tract-related AEs may have been biased by the participant's first regimen assignment and/or previous exposure to cervical barriers. The study's randomized, crossover design controlled for any possible bias towards the regimen assigned first or second. We purposively selected an equal number of women with and without previous diaphragm experience, and stratified our adherence and acceptability results by this characteristic (few women reported recent diaphragm use at baseline, so this was not done for the safety results).

This study was short in duration, and it is difficult to predict how acceptability and safety outcomes might change with longer use time. In this analysis we included only quantitative, pre-designated measures of product acceptability, rather than our qualitatively captured data. This approach potentially limits a broader understanding of product acceptability, and important reasons for liking/disliking Duet may have been overlooked.

Acceptability, adherence and safety measures, with the exception of clinical observation of AEs, relied on self-report, which may have been subject to social desirability and recall bias. We minimized this bias by measuring adherence and acceptability in several ways, and found high concordance between multiple measures (i.e., proportion of acts/days covered and the opt-in selection of "Not applicable, I used it every time" when asked about non-use).

Further, participants were not excluded for non-adherence or for disliking the products, and most AEs that might have warranted discontinuation would have been observed during pelvic exams. Nonetheless, the level of condom use reported in this sample of married women on effective contraception was high, although similar to other research in this setting [15,30,12], and it is unknown whether this is from social desirability bias or from the successful implementation of intensive condom counselling in this study, and/or previous research studies. Related to this final point, it is also important to acknowledge that the women able and willing to join this study, and meeting entry criteria, may not have been representative of the general population, nor of those at highest risk of HIV/STI acquisition.

## Conclusions

We found that the Duet was highly acceptable and safe for use both continuously and precoitally in this African setting when used for 14-day periods. Further research should explore the safety of the device with alternative microbicide candidates as a low-cost, reusable delivery mechanism and potential disease prevention option. In this study, assignment to continuous-use regimen did not equate with more protected sex acts. However, the strategy may be more effective in enabling adherence over periods of time longer than 14 days. Users familiar with precoital insertion of cervical barriers or other methods might require more counselling to encourage continuous use.

## Competing interests

TRM is the inventor and manufacturer of the Duet device and BufferGel. No other authors hold competing interests.

## Authors' contributions

ETM participated in the development of the study protocol, data collection instruments and procedures, monitored field operations and data, led the analysis, and drafted the manuscript. CW conceived of the study, developed the protocol and instruments, oversaw field implementation, and reviewed and edited the manuscript. PM coordinated and managed the data collection, and participated in the development of instruments and procedures. HC performed the statistical analysis. TC oversaw the field site, provided clinical expertise to the protocol development, and attended to all clinical and safety-related issues, as applicable. TRM participated in the development and design of the project and protocol, provided clinical input on data, and reviewed and edited the manuscript. FS provided clinical technical assistance in the oversight of safety issues, and reviewed and edited the manuscript. AVDS participated in the development and design of the project and protocol, provided technical expertise in the development and interpretation of behavioural assessments, and reviewed and edited the manuscript. All co-authors read and approved the final version of the manuscript.
